# The Z Mutation Alters the Global Structural Dynamics of α_1_-Antitrypsin

**DOI:** 10.1371/journal.pone.0102617

**Published:** 2014-09-02

**Authors:** Victoria A. Hughes, Robert Meklemburg, Stephen P. Bottomley, Patrick L. Wintrode

**Affiliations:** 1 Department of Biochemistry and Molecular Biology, Monash University, Clayton, Victoria, Australia; 2 Department of Pharmaceutical Sciences, University of Maryland School of Pharmacy, Baltimore, Maryland, United States of America; INRA, France

## Abstract

α_1_-Antitrypsin (α_1_AT) deficiency, the most common serpinopathy, results in both emphysema and liver disease. Over 90% of all clinical cases of α_1_AT deficiency are caused by the Z variant in which Glu342, located at the top of s5A, is replaced by a Lys which results in polymerization both *in vivo* and *in vitro*. The Glu342Lys mutation removes a salt bridge and a hydrogen bond but does not effect the thermodynamic stability of Z α_1_AT compared to the wild type protein, M α_1_AT, and so it is unclear why Z α_1_AT has an increased polymerization propensity. We speculated that the loss of these interactions would make the native state of Z α_1_AT more dynamic than M α_1_AT and that this change renders the protein more polymerization prone. We have used hydrogen/deuterium exchange combined with mass spectrometry (HXMS) to determine the structural and dynamic differences between native Z and M α_1_AT to reveal the molecular basis of Z α_1_AT polymerization. Our HXMS data shows that the Z mutation significantly perturbs the region around the site of mutation. Strikingly the Z mutation also alters the dynamics of regions distant to the mutation such as the B, D and I helices and specific regions of each β-sheet. These changes in global dynamics may lead to an increase in the likelihood of Z α_1_AT sampling a polymerogenic structure thereby causing disease.

## Introduction

**Figure 1:**
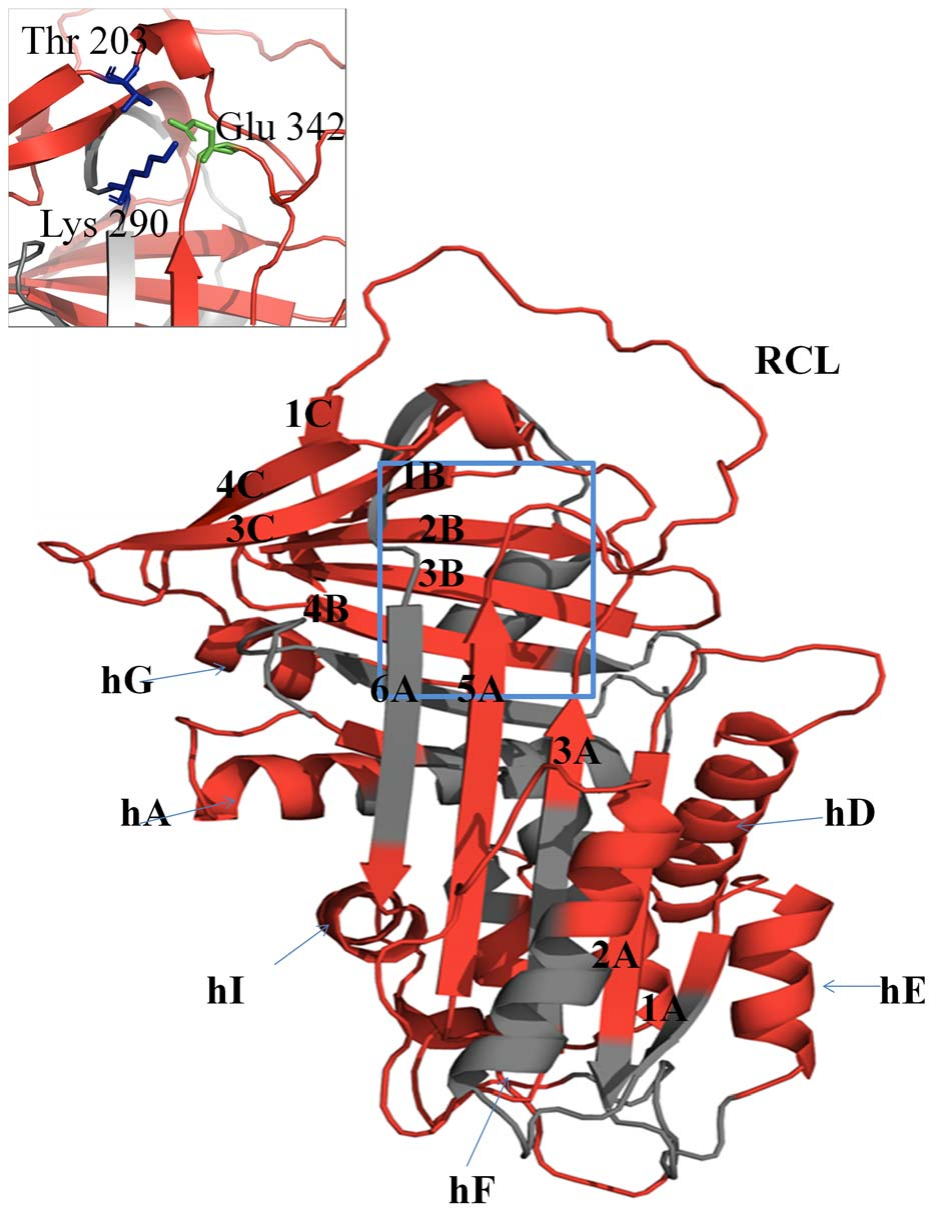
The structure and sequence of α_1_AT. (A) Ribbon diagram of M α_1_AT (PDB: 1QLP) [24] is shown and the peptic fragments used in this study are highlighted in red. The insert shows a close up view of the region around Glu342, the site of the Z mutation. Figures are prepared using PyMol (2002). The PyMOL Molecular Graphic System, San Caros, CA, U.S.A.).

The misfolding and subsequent polymerization of members of the serpin superfamily leads to a variety of diseases collectively known as the Serpinopathies [1]. The most common serpinopathy is α_1_-antitrypsin (α_1_AT) deficiency, which affects approximately 1 in 2000 people [2]. The serpin, α_1_AT, is synthesized by hepatocytes and released into the circulation where it protects the lung from the action of neutrophil elastase. Over 70 mutations have been identified that lead to α_1_AT deficiency. The most common pathological variant, accounting for 95% of all clinical cases, is the Z variant [3], [4], [5] in which Glu342, which is located at the junction between the top of s5A and the base of the reactive center loop (RCL), is replaced by a Lys (Fig. 1a). The presence of this mutation results in the removal of both a salt bridge to Lys290 and a hydrogen bond to Thr203. The loss of these interactions brings about misfolding and polymerization of the protein within the endoplasmic reticulum of hepatocytes resulting in a lack of secretion and is characterized by a reduction in plasma levels to 10–15% of normal [6]. The polymerized Z α_1_AT damages the hepatocytes and predisposes the carrier to liver disease. The decreased plasma levels give rise to severe early onset emphysema.

The molecular basis of Z α_1_AT polymerization is not completely understood. The structure, stability and polymerization characteristics of native Z α_1_AT have been studied using a range of biochemical and biophysical techniques [4], [7], [8], [9]. These data reveal that Z α_1_AT, in contrast to wild type α_1_AT (M α_1_AT), polymerizes rapidly when incubated at physiological temperatures [4], [6], [10]. The crystal structure of Z α_1_AT has not yet been determined however it is still an efficient proteinase inhibitor indicating that it possesses the serpin fold [9], [10]. In support of this the equilibrium unfolding of Z α_1_AT has been studied and shown to be the same as M α_1_AT suggesting that compensating interactions are formed in Z α_1_AT to counteract for the loss of the two native state interactions [8], [11]. Two additional pieces of experimental evidence suggest that there are substantial differences within the native state of Z α_1_AT. First, recent spectroscopic data using mutants of M and Z α_1_AT have shown that there are structural differences between the proteins [8], [11]. Secondly, kinetic unfolding studies indicated that in the three state unfolding reaction the transition from the native state to a partially folded intermediate state proceeds almost two times faster for Z α_1_AT than for M α_1_AT [4]. Therefore, we speculated that the native state of Z α_1_AT may be more dynamic than M α_1_AT and that it is this change which renders the protein prone to polymerization. To examine this hypothesis we have used hydrogen/deuterium exchange combined with mass spectrometry (HXMS) to determine the structural and dynamic differences between native Z and M α_1_AT and to reveal the molecular basis of Z α_1_AT polymerization.

## Results

M and Z α_1_AT appear to possess similar thermodynamic stability [4], [8], yet native Z α_1_AT, incubated at physiological temperatures (37–41°C), readily polymerizes whereas M α_1_AT does not [6], [8], [9]. One potential explanation for the rapid polymerization of Z α_1_AT is that it is in, or can access more readily, a non-native, yet active, conformation [12], [13]. In order to examine this possibility we compared the native state dynamics of both M and Z α_1_AT using HXMS coupled with pepsin digestion, to measure the flexibility of specific regions within these serpins [14].

Both M and Z α_1_AT were expressed in *P. pastoris* and purified as previously described [10]. The H/D exchange of the M α_1_AT used in this study is in excellent agreement with our previous study using M α_1_AT produced in *E. coli* [15]. Tandem mass spectrometry experiments were carried out and 132 overlapping peptic fragments were identified from both M and Z α_1_AT. A comparison of H/D exchange of native M and Z α_1_AT was performed at pD 8 and 25°C followed by pepsin digestion and HPLC-MS to quantify the mass of each peptic fragment. Analysis of the pepsin digest of undeuterated M and Z α_1_AT under the rapid HPLC gradient required for the H/D experiments identified 19 peptic fragments, with good signal to noise ratio (Table 1). These fragments cover 79% of the entire α_1_AT molecule and are well distributed throughout the sequence; the only significant gaps in coverage encompass regions around helices A and H (Fig. 1a,b).

**Table 1:** Details of the peptides derived from pepsin digestion and tandem mass spectrometry experiments.

Residue Number	Secondary structure elements	Amino acid sequence (Full stop indicates digestion site)	MW	Z	MH^2+^
38–51	hA-β6B	L.YRQLAHQSNSTNI.F	1530.75	2.00	765.875
38–62	hA-β6B	L.YRQLAHQSNSTNIFFSPVIATA.F	2464.24	2.00	1232.62
62–77	hB-hC	F.AMLSLGTKADTHDEIL.E	1714.87	2.00	857.93
81–100	hD	N.FNLTEIPEAQIHEGFQEL.L	2115.04	2.00	1058.02
101–119	hD-β2A	L.LRTLNQPDSQLQLTTGNGLF.L	2216.17	2.00	1108.58
127–142	hE-β1A	L.VDKFLEDVKKLYHSEA.F	1921.01	2.00	961.00
160–172	hF-loop	D.YVEKGTQGKIVDL.V	1449.79	2.00	725.40
171–182	loop	D.LVKELDRDTVF.A	1334.73	2.00	667.87
191–212	Loop	G.KWERPFEVKDTEE.E	1691.82	2.00	845.91
215–227	β4C-β3C	F.HVDQVTTVKVPMMKRLGMF.N	2217.17	2.00	1109.09
227–240	β1B-β2B	F.NIQHCKKLSSWVL.L	1555.84	2.00	778.42
240–252	β2B-β3B	L.LMKYLGNATAIF.F	1341.72	2.00	671.36
252–272	hG-hH	F.FLPDEGKLQHLENELTHD.I	2135.04	2.00	1068.02
297–303	hI	T.YDLKSVL.G	837.47	1.00	
304–317	Hi-loop	L.GQLGITKVFSNGAD.L	1406.73	2.00	703.86
325–338	β5A	E.APLKLSKAVHKAVL.T	1474.95	2.00	737.97
339–353	RCL	L.TIDKKGTEAAGAMFL.E	1552.80	2.00	776.90
353–372	RCL- β1C- β4B	L.EAIPMSIPPEVKFNKPFVF.L	2190.17	2.00	1095.58
372–384	β4B- β5B	F.LMIEQNTKSPLF.M	1420.75	2.00	710.88

The relative masses used in this study were determined using Sequest.

**Figure 2:**
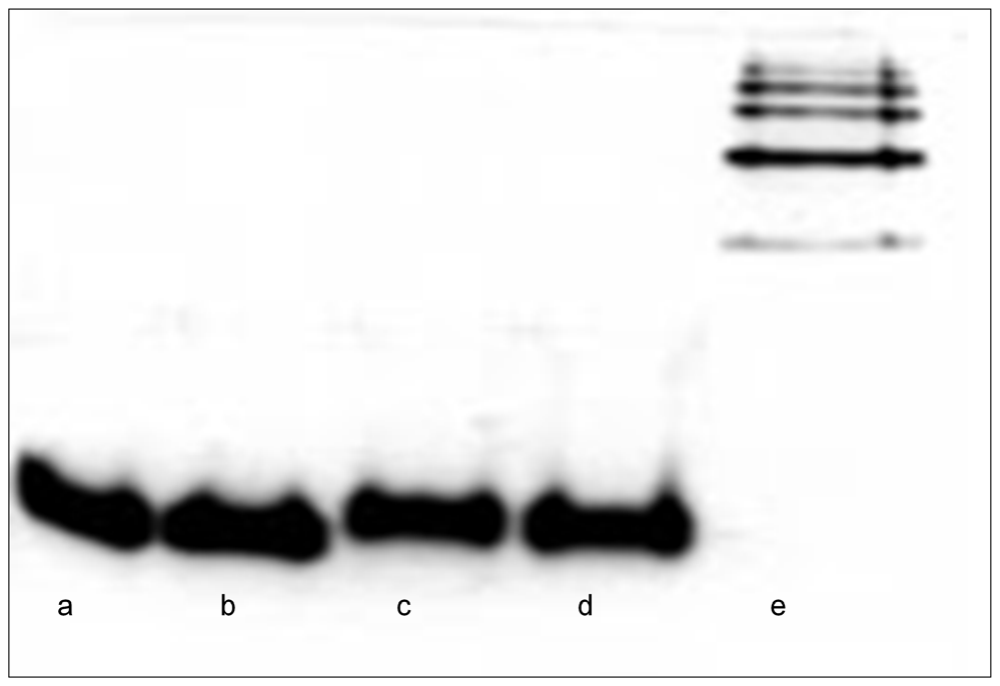
Native Page analysis of M and Z α_1_AT under HDX conditions. M and Z α_1_AT were incubated in D_2_O buffered with 10 mM Tris (pD 8) at 25°C for up to 2500 seconds. Samples of the proteins were then analyzed by 10% Native PAGE. (A): M α_1_AT t = 0 seconds; (B) M α_1_AT t = 2500 seconds; (C) Z α_1_AT t = 0 seconds; (D) Z α_1_AT t = 2500 seconds and (E) Z α_1_AT polymers purified directly from *P. Pastoris* 10].

Using previously established procedures in our laboratory [15], [16] we were able to measure the kinetics of deuterium incorporation for the 19 peptic fragments (Table 1), from both M and Z α_1_AT over a period of 2000 sec. The first experimental point measured was 10 sec after isotope exchange was initiated. Deuterium labelling was performed at 25°C, with deuteration times ranging from 10 to 2000 s. Under these experimental conditions, both M and Z α_1_AT remained in a monomeric form during the deuterium labelling time (Fig. 2).

**Figure 3:**
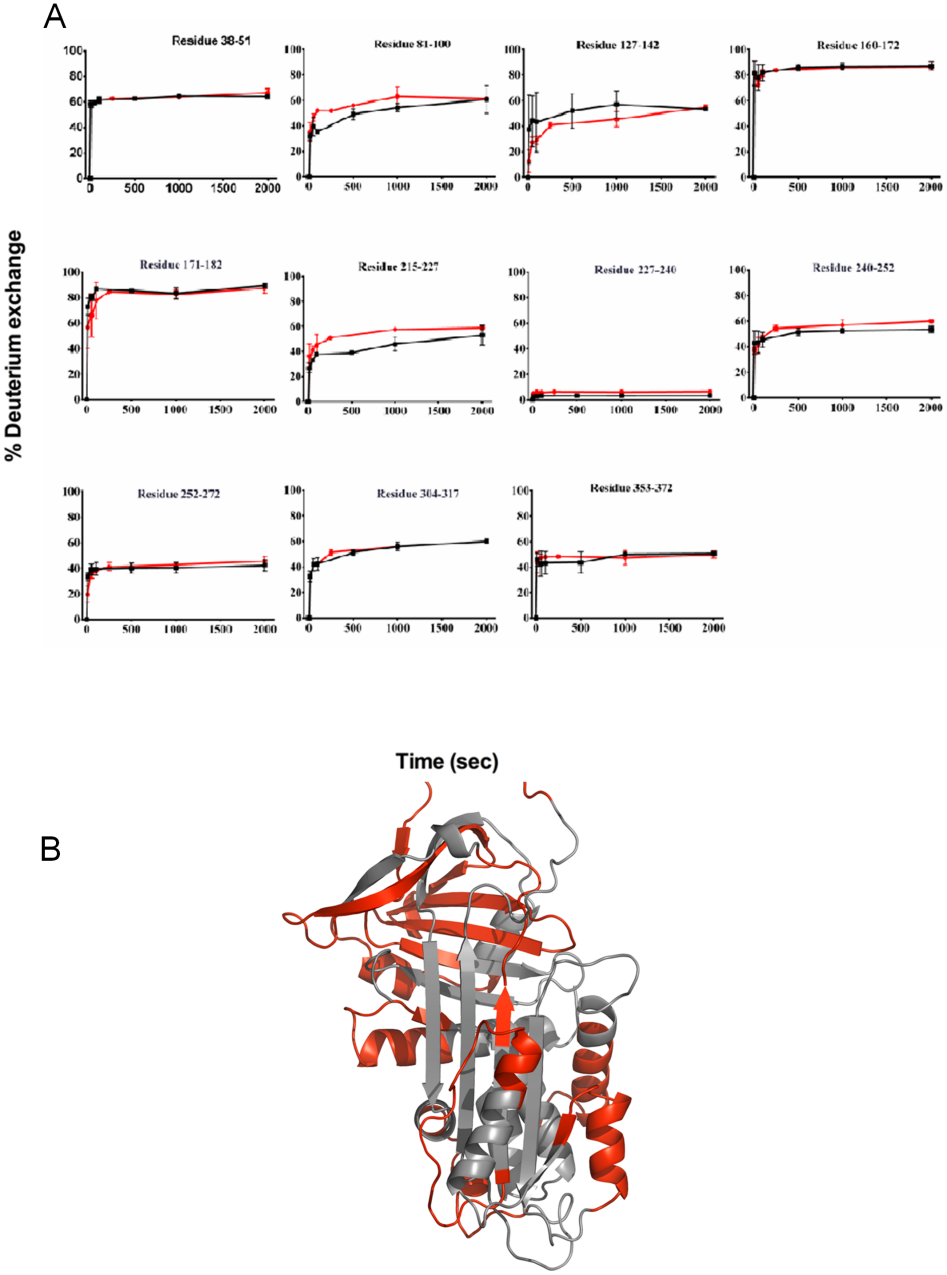
Peptic fragments derived from α_1_AT that show comparable exchange kinetics in M and Z α_1_AT. (A) The kinetics of deuterium incorporation into M α_1_AT (black) and Z α_1_AT (red) by individual peptic fragments which show comparable exchange are shown. The individual data points are the average of three independent experiments for clarity the error bars are not shown. (B) Crystal structure of M α_1_AT (PDB: 1QLP [24]) indicating the location of peptic fragments with comparable exchange highlighted in red.

Twelve pairs of peptides from M and Z α_1_AT displayed similar kinetics and extent of deuterium incorporation. These data therefore suggest that the Z mutation had minimal structural or dynamic effects on the serpin in these regions which are spread throughout the molecule (Fig. 3).

**Figure 4:**
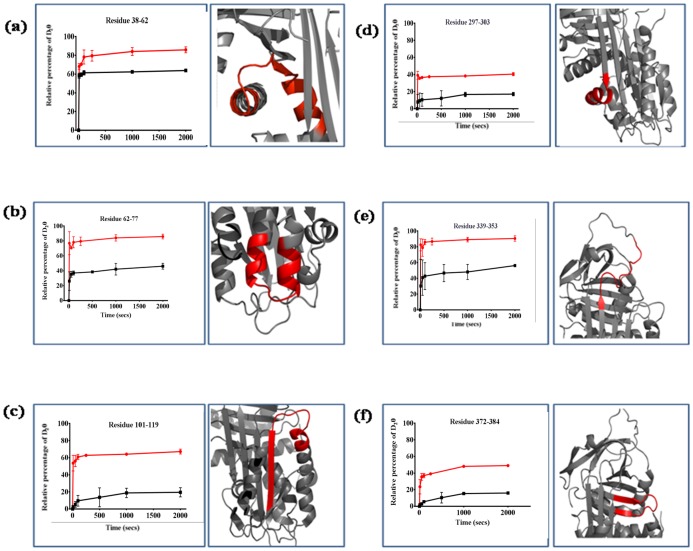
Peptic fragments derived from α_1_AT that display enhanced deuterium incorporation in Z α_1_AT. The kinetics of deuterium incorporation into M α_1_AT (black) and Z α_1_AT (red) of peptic fragments which show significant increased deuterium uptake in Z compared to M α_1_AT. A close up view of the location of the peptide fragment (red) within α_1_AT (PDB: 1QLP)[24] is shown. The individual data points are the average of three independent experiments for clarity the error bars are not shown.

Six peptides showed a significant enhancement of deuterium exchange in Z α_1_AT compared to M α_1_AT (Fig. 4a–f). The extent of labelling was increased for peptic fragments 38–62 (hA-hB), 62–77 (hB-hC), 101–119 (β2A), 297–303 (β6A-hI), 339–353 (β5A-Linker) and 372–384 (β4B-β5B). Results for these peptic fragments were mapped onto the crystal structure of M α_1_AT with the peptides showing an increase in exchange of the peptide in Z α_1_AT compared to M α_1_AT coloured red (Fig. 4a–f).

**Figure 5:**
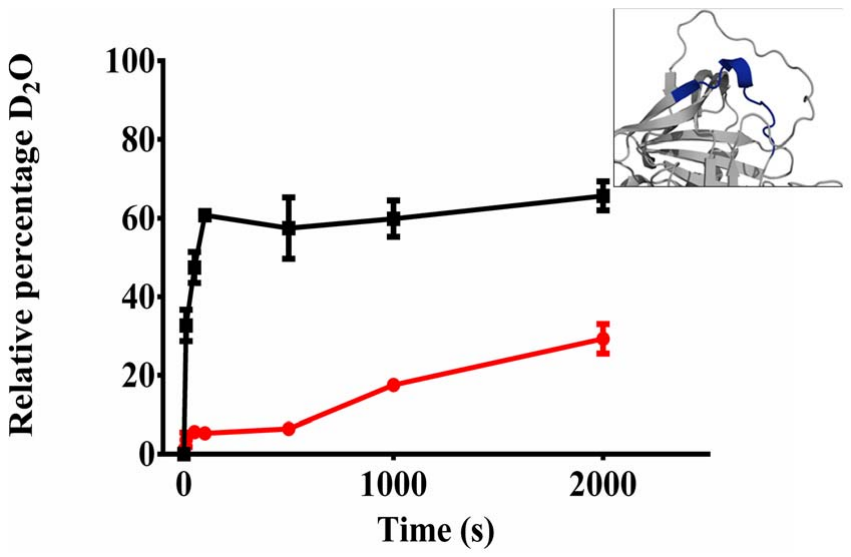
Peptic fragments derived from α_1_AT that display enhanced deuterium incorporation in M α_1_AT. (A) The kinetics of deuterium incorporation into M α_1_AT (black) and Z α_1_AT (red) of peptic fragments which show significant increased deuterium uptake in M compared to Z α_1_AT. Insert- Crystal structure of M α_1_AT (PDB: 1QLP) [24] indicating the location of peptic fragments with decreased exchange highlighted in blue. The individual data points are the average of three independent experiments for clarity the error bars are not shown.

Only one peptide, 191–212, (the loop connecting β3A and β4C) showed a significant reduction of deuterium exchange in Z α_1_AT compared to M α_1_AT (Fig. 5a,b). Results for this peptide was mapped onto the structure of M α_1_AT with the peptide showing an increase in exchange in M α_1_AT compared to Z α_1_AT coloured in blue (Fig. 5b).

**Figure 6:**
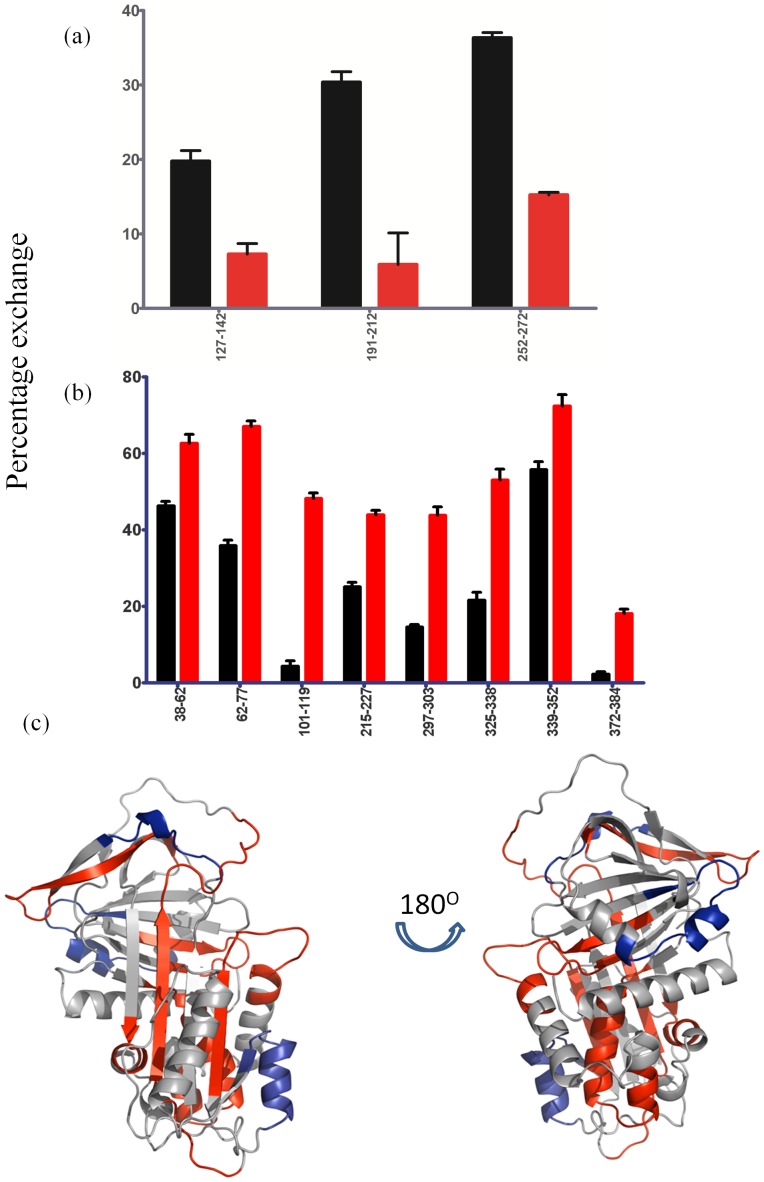
Regions in α_1_AT that are affected by the Z mutation. (A) Peptides that displayed decreased exchange in Z α_1_AT (Red) compared to M α_1_AT (Black); (B) Peptides that displayed enhanced exchange in Z α_1_AT (Red) compared to M α_1_AT (Black) at 10 sec. (C) The structure of α_1_AT (PDB: 1QLP) [24] indicating residues with an increased D_2_O uptake in Z (red) and decreased D_2_O uptake in Z (Blue) after 10 seconds of incubation in D_2_O.

Three peptic fragments, 127–142 (hE-β1A), 191–212 (β3A-β4C) and 252–272 (β3B-hG-hH), displayed greater than two-fold protection in Z α_1_AT in comparison to M α_1_AT after only 10 seconds of deuterium labelling (Fig. 6a). Under these conditions, amides in unfolded regions of the molecule will undergo nearly complete exchange, while hydrogens in folded regions remain largely unexchanged. This type of pulse labelling has been shown to be an effective tool for monitoring site specific folding and unfolding in proteins [17]. The hydrogens in these 3 peptides are less labile due to decreased flexibility or a different conformation of the peptide in Z α_1_AT. It is also clear that there is considerable exchange in peptic fragments 38–62 (β6B-hB), 62–77 (hB-hC), 101–119 (hD-β2A), 215–227 (β3C) 297–303 (β6A-hI), 325–338 (β5A), 339–353 (β5A-Linker) and 372–384 (β4B-β5B) (Fig. 6B). These data suggest that regions covered by these peptides are either partially unfolded or marginally stable in Z α_1_AT.

Significant differences in deuterium incorporation are also observed at longer exchange times and suggest that globally Z α_1_AT is more dynamic than M α_1_AT. To better represent the data we have grouped the deuterium exchange into classes depending on the exchange at 2000 seconds. Class 1 peptides exchange rapidly in the native state with greater than 80% exchange in 2000 seconds. Peptic fragments 62–77 encompassing the helices B–C show rapid exchange in Z α_1_AT only, suggesting a lack of stable secondary structure leading to a more dynamic molecule. The rapid exchange of the peptic fragments 339–352 and 352–372 corresponding to the RCL show an enhanced exchange suggesting less interactions in Z than WT α_1_AT.

Class 2 peptides show moderate exchange (30–80%) at 2000 seconds in the native state and are shown in yellow. Residues within areas of high α-helical and β-sheet content are expected to exchange more slowly than those of turns and loops and make up the majority of peptides seen for class 2 [18]. Peptic fragments 38–51, 127–142, and 304–317 associated with helix D, E, G and I respectively show only a 60% exchange at 2000 seconds in M and Z α_1_AT. Residues 191–212 (the loop connecting β3A and β4C) also fall into the category although this is the only peptide that shows a reduction in exchange in Z α_1_AT.

**Figure 7:**
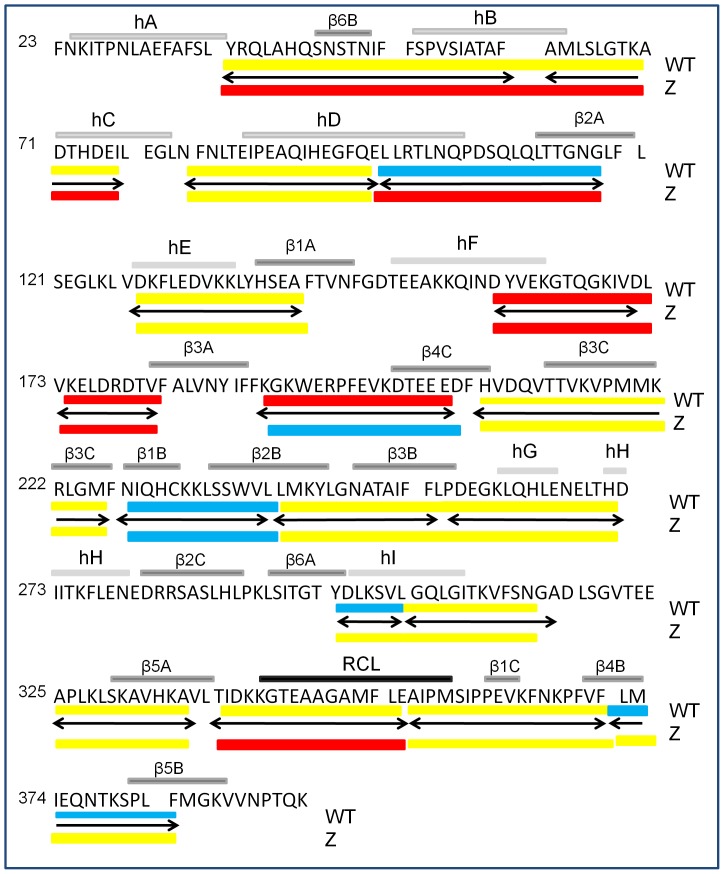
Differences in hydrogen exchange at 2000 seconds between M and Z α_1_AT. The amino acid sequence of α_1_AT is shown with secondary structure highlighted above the sequence. The 18 peptides used in the study are noted, as double headed arrows. The peptides for both M and Z α_1_AT are colored according to the percentage deuterium incorporation at 2000 seconds: class 1 80–100% (red), class 2 30–80% (yellow) and class 3 0–30% (blue).

In M α1AT, peptic fragments that are protected from exchange include the top of hD and β2A, , β1B-β2B,, β6A-hI and the loop connecting β4B to β5B, (101–119, , 227–240, 297–303 and 372–384) previously attributed to the hydrophobic core [15], [19] and are described as showing class 3 exchange in yellow in figure 7. Z α1AT shares with M α1AT only residues (227–240) that show significant protection and differently from M protein displays residues 191–212 having high protection as discussed before, both peptides belonging to class 3.

## Discussion

The structural integrity of a protein generally relies on its ability to adopt and maintain a unique native state. For members of the serpin superfamily the integrity of the native state must also allow local motions that facilitate proteinase inhibition. However, these motions can be highjacked and used to promote disease causing polymerization. In the case of Z α_1_AT we have a protein whose fold and apparent thermodynamic stability are similar to M α_1_AT, yet it polymerizes from the native state much more rapidly [4]. Using HDX we have examined the global and local changes that arise in the natively folded ensembles of Z α_1_AT, this study shows that previous studies may have underestimated the effect of the E242K substitution on the molecule and the effects are not just localised at the site of mutation but extend to distant regions of the structure.

The HDX results presented here reveal that the structural effects due to the E342K mutation are not distributed uniformly throughout the structure, but are instead localized in specific regions. Exchange at 10 seconds indicates partial loss of structure in several regions, the most dramatic being β2A and the top of hD (Fig. 6a). Compared with M α_1_AT, Z α_1_AT has lost ∼8 hydrogen bonds in this region, suggesting significant disruption of interactions between β2A and the surrounding structural elements. Previous molecular dynamics simulations support the idea that the effects of the Glu342Lys substitution can propagate to this region. While significant disruption of β2A was not observed on the 50 ns timescale to the simulations, a large change in the conformation of the hD-β2A loop was observed, consistent with our HDX results [11]. The top of helix F remains highly dynamic as previously seen in M α_1_AT [15], [19]. Deuterium levels at 10 s also indicate that the region covered by residues 339–353 has lost ∼3 hydrogen bonds, suggesting a loss of structure at the top of β-sheet A that is an important site in the early stages of RCL insertion. Additionally, there is disruption of hydrogen bonds between the central portion of β3A and the adjacent β2A and β5A. Loss of hydrogen bonds in these regions, together with smaller but still significant losses in helices A, B, and C, clearly demonstrates that the E342K mutation disrupts native structure in areas both distant from and close to the mutation site. In addition to the loss of hydrogen bonds, deuterium uptake at 10 seconds also indicates the formation of additional hydrogen bonds in regions spanned by residues 127–142, 191–212 and 252–272, in Z α_1_AT compared to M. These regions correspond to hE-β1A, β3A-β4C and hG-hH respectively. However, the added hydrogen bonds do not appear to be stable, as the additional protection against deuterium uptake in Z α_1_AT is lost within 100 seconds (for peptides 191–212 and 352–372) to 1000 seconds (for peptide 191–212). Taken together these results on deuterium uptake at 10 seconds clearly indicate that Z α_1_AT exists in an altered native conformation compared to M α_1_AT and that there is significant disruption of hydrogen bonding in much of β-sheet A which is in agreement with our previously published data using site single point mutations and molecular dynamic simulations [11] [8].

Significant differences in the extent of deuterium exchange at longer labeling times were found within 7 peptides (Fig. 4 and 5), indicating dynamic and structural differences between the two proteins. One of the peptides (residues 339–353, the top of s5A and the RCL) includes the mutation site, Glu 342; this peptide was observed to be more mobile in Z α_1_AT (Fig. 4e). Also in this region was peptide 191–212 (β3A-β4C) which displayed decreased deuterium uptake indicating that this region contains additional hydrogen bonds and is more rigid in Z α_1_AT (Fig. 5). This increased rigidity may be due to stabilizing interactions between Lys342 and Glu199. Trp194 is located in this region, and the increased rigidity may appear to be at odds with previous results showing differences in Trp fluorescence between M and Z α_1_AT. We note, however, that while the region covered by the peptide containing Trp194 shows decreased exchange at short times, the top of β5A, which is immediately adjacent to Trp194, shows increased exchange, indicating a more dynamic local environment. We therefore conclude that there is no inconsistency between the fluorescence and H/D exchange data. These changes in deuterium uptake suggests that the interactions within the vicinity of the mutation are altered by the removal of the salt bridge between Glu342 and K290, which allows this region to sample a conformation in which the top of s5A is open. This open conformation is maintained by new interactions formed between Lys342 and Val200, Thr203 present within peptide 191–212 [11].

There are several regions, distant from the mutation site, whose structure and stability depend upon the residues they pack against such as helix A, B and H which are affected by the Z mutation [20], [21]. We observe a significant increase in the flexibility of peptic fragments corresponding to the helix B in Z α_1_AT (Fig. 4a, b). Peptide 38–51 show a comparable behavior in both M and Z α_1_AT (Fig. 3a) whereas an increase in exchange is seen for residues 38–62 (Fig. 4a) suggesting that the increase in exchange can be attributed to the B. The flexibility in this region suggests that the amide hydrogen bonds in these peptides are less stable and the packing around the helix is loosened in Z relative to M α_1_AT and may explain the loss of helical structure seen in the CD spectra of Z α_1_AT [4], [9].

**Figure 8:**
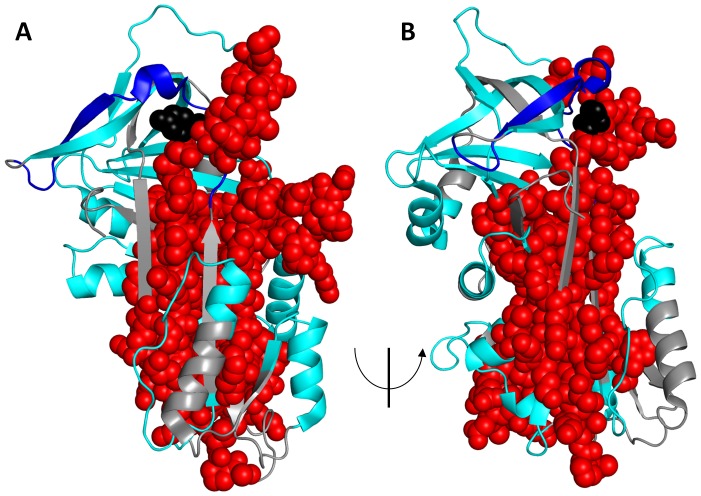
Summary of differences in HDX between M and Z α_1_AT. The structure of α_1_AT (PDB: 1QLP) [24] with Red spheres: regions showing increased HDX in Z. Dark blue: regions showing decreased HDX in Z. Cyan: regions showing no significant difference between M and Z. Grey: regions for which no peptides were analyzed.

What is apparent from the experimental data is reduced protection in the hydrophobic core of Z α_1_AT (Figs. 4 and 6). In fact, regions showing significantly increased exchange in Z α_1_AT form a nearly contiguous group that encompasses much of the core of the molecule (Figure 8). The exchange resistant core of M α_1_AT has previously been shown to consist of β-sheet rich regions [15], [16]. In M α_1_AT, peptic fragments that are protected from exchange include β2A, β3A-β4C, β2B-β3B and β6A-hI, (101–119, 191–212, 227–240, 297–303) and are described as showing class 3 exchange in figure 7. For Z α_1_AT only residues (227–240) show significant protection with residues corresponding to β2A, β3A-β4C, β2B-3B and β6A-hI demonstrating class 2 behaviour (Fig. 7). The peptide covering residues 227–240 are heavily protected from exchange in both M and Z α_1_AT with less than 10% of the hydrogen available for exchange exchanging in the experimental time frame. This peptide, which has been identified in several previous studies as being resistant to chemical denaturation [22], [23] and has been proposed to play a role as folding initiator [23], remains unaffected by the Z mutation. The increased deuterium exchange seen for the ‘core’ peptides in Z α_1_AT may allow the molecule to sample conformations that on the folding pathway and lead to accumulation of the polymerogenic folding intermediate that the open sheet intermediate is on-pathway and the loss of the salt bridge leads to enhanced lability and ability to switch to a polymerogenic conformation.

In conclusion, our data clearly demonstrates that the single mutation Glu342Lys results in global dynamic changes to the serpin fold. This in turns leads to an increase in the probability of Z α_1_AT sampling an open sheet structure capable of polymerisation.

## Materials and Methods

### Expression and purification of M and Z α_1_AT

M and Z α_1_AT were expressed and purified from *P. Pastoris* as previously described [10].

### Peptide mapping by high performance liquid chromatography (HPLC)-Tandem mass spectrometry

Peptide mapping was carried out as previously described [15]. In brief, a total of 5 µg (0.1 nmol) of purified M or Z α_1_AT in 100 µL of 50 mM Tris (pH 8) and 50 mM NaCl was mixed with 95 µL of 100 mM NaH_2_PO_4_ (pH 2.4) followed by the addition of 5 µg of porcine pepsin dissolved in 0.05% (v/v) TFA and H_2_O for pepsin digestion. M or Z α_1_AT was digested for 5 min on ice. The digested sample was then injected into a micropeptide trap (Michrom Bioresources) connected to a C18 HPLC column (5 cm×1 mm, Alltech) coupled to a LTQ linear ion-trap mass spectrometer (ThermoElectron). Peptic fragments were eluted using a gradient of acetonitrile (Burdick and Jackson) at a flow rate of 50 µL/min for a tandem mass spectrometry experiment to sequence each peptic fragment. Peptic fragments were identified by using the search algorithm SEQUEST (ThermoElectron) and manual inspection.

### Hydrogen/Deuterium Exchange

A sample containing 5 µg (0.1 nmol) of M or Z α_1_AT in 50 mM Tris (pH 8) and 50 mM NaCl was diluted 24-fold with 50 mM Tris and 50 mM NaCl dissolved in D_2_O (Cambridge Isotope Laboratories) at 25°C to label the sample. The deuteration reaction was quenched at different time points by adding an equal volume of 100 mM NaH_2_PO_4_ (pH 2.4) and quickly frozen in a dry ice−ethanol bath. Samples were stored at −80°C until use.

### Isotope Analysis by HPLC−Electrospray Ionization Mass Spectrometry (ESI-MS)

The frozen sample was quickly thawed and digested with 5 µg of pepsin on ice for 5 min followed by immediate injection into a micropeptide trap connected to a C18 HPLC column coupled to a Finnigan LCQ quadrupole ion-trap mass spectrometer. Peptic peptides were eluted in 12 min using a gradient of 10–45% acetonitrile at a flow rate of 50 µL/min. The micropeptide trap and C18 HPLC column were immersed in ice to minimize back exchange. Because the mass of a peptic fragment increases by one for every amide hydrogen atom exchanged with deuterium, the amount of deuterium in each peptic fragment can be determined by comparing the mass of a labelled peptic fragment with the mass of the same peptide without the label. The centroid mass of each peptic fragment was determined using the software package MagTran. To correct for the back-exchange reaction of hydrogen atoms during pepsin digestion and HPLC−MS, a fully deuterated sample was prepared by incubating 5 µg of M or Z α_1_AT in 6 M guanidine deuterochloride, 50 mM Tris (pH 8) and 50 mM NaCl for 60 min at 25°C. The deuterium incorporation of each peptic fragment, corrected for the back exchange, was calculated using the following equation: D/N = [(*m−m_0%_)/(m_100%_−m*)] where *m* is the mass of deuterated peptic fragment, *m*_0%_ and *m*_100%_ are the mass of the unlabeled and fully deuterated peptic fragments, respectively, *N* is the total number of exchangeable amide hydrogen atoms in each peptic fragment, and *D* is the number of amide hydrogen atoms incorporated in each peptic fragment.

## References

[1] Gooptu B, Lomas DA (2009) Conformational Pathology of the Serpins: Themes, Variations, and Therapeutic Strategies. Ann Rev Biochem 78: 147–176. 19245336 10.1146/annurev.biochem.78.082107.133320

[2] Blanco I, Fernández E, Bustillo EF (2001) Alpha-1-antitrypsin PI phenotypes S and Z in Europe: an analysis of the published surveys. Clin Gen 60: 31–41. 10.1034/j.1399-0004.2001.600105.x 11531967

[3] Knaupp AS, Bottomley SP (2009) Serpin Polymerization and Its Role in Disease-The Molecular Basis of alpha(1)-Antitrypsin Deficiency. Iubmb Life 61: 1–5. 18785256 10.1002/iub.127

[4] Knaupp AS, Levina V, Robertson AL, Pearce MC, Bottomley SP (2010) Kinetic Instability of the Serpin Z [alpha]1-Antitrypsin Promotes Aggregation. J Mol Biol 396: 375–383. 19944704 10.1016/j.jmb.2009.11.048

[5] Fregonese L, Stolk J (2008) Hereditary alpha-1-antitrypsin deficiency and its clinical consequences. Orphanet Journal of Rare Diseases 3: 16. 18565211 10.1186/1750-1172-3-16 PMC2441617

[6] Lomas DA, Evans DL, Finch JT, Carrell RW (1992) The mechanism of Z alpha 1-antitrypsin accumulation in the liver. Nature 357: 605–607. 1608473 10.1038/357605a0

[7] Yu MH, Lee KN, Kim J (1995) The Z type variation of human alpha 1-antitrypsin causes a protein folding defect. Nat Struct Biol 2: 363–367. 7664092 10.1038/nsb0595-363

[8] Knaupp AS, Bottomley SP (2011) Structural Change in B-Sheet A of Z1-Antitrypsin Is Responsible for Accelerated Polymerization and Disease. J Mol Biol 413: 888–898. 21945526 10.1016/j.jmb.2011.09.013

[9] Lomas DA, Evans DL, Stone SR, Chang WS, Carrell RW (1993) Effect of the Z mutation on the physical and inhibitory properties of alpha 1-antitrypsin. Biochem 32: 500–508. 8422359 10.1021/bi00053a014

[10] Levina V, Dai WW, Knaupp AS, Kaiserman D, Pearce MC, et al (2009) Expression, purification and characterization of recombinant Z alpha(1)-Antitrypsin-The most common cause of alpha(1)-Antitrypsin deficiency. Prot Exp and Purification 68: 226–232. 10.1016/j.pep.2009.06.011 19555763

[11] Kass I, Knaupp AS, Bottomley SP, Buckle AM (2012) Conformational properties of the disease-causing Z variant of alpha1-antitrypsin revealed by theory and experiment. Biophys J 102: 2856–2865. 22735536 10.1016/j.bpj.2012.05.023 PMC3379022

[12] Mahadeva R, Dafforn TR, Carrelll RW, Lomas DA (2002) 6-mer peptide selectively anneals to a pathogenic serpin conformation and blocks polymerization - Implications for the prevention of Z alpha(1)-antitrypsin-related cirrhosis. Journal of Biological Chemistry 277: 6771–6774. 11773044 10.1074/jbc.C100722200

[13] Chang WS, Wardell MR, Lomas DA, Carrell RW (1996) Probing serpin reactive-loop conformations by proteolytic cleavage. J Biol Chem 314: 647–653. 10.1042/bj3140647 PMC1217096 8670081

[14] Wales TE, Engen JR (2006) Hydrogen exchange mass spectrometry for the analysis of protein dynamics. Mass Spectrometry Reviews 25: 158–170. 16208684 10.1002/mas.20064

[15] Tsutsui Y, Liu L, Gershenson A, Wintrode PL (2006) The conformational dynamics of a metastable serpin studied by hydrogen exchange and mass spectrometry. Biochem 45: 6561–6569. 16716066 10.1021/bi060431f

[16] Tsutsui Y, Kuri B, Sengupta T, Wintrode PL (2008) The Structural Basis of Serpin Polymerization Studied by Hydrogen/Deuterium Exchange and Mass Spectrometry. J Biol Chem 283: 30804–30811. 18794298 10.1074/jbc.M804048200 PMC2576545

[17] Deng Y, Smith DL (1999) Rate and Equilibrium Constants for Protein Unfolding and Refolding Determined by Hydrogen Exchange-Mass Spectrometry. Anal Biochem 276: 150–160. 10603237 10.1006/abio.1999.4347

[18] Bai Y, Milne JS, Mayne L, Englander SW (1993) Primary structure effects on peptide group hydrogen exchange. Proteins: Structure, Function, and Genetics 17: 75–86. 10.1002/prot.340170110 PMC3438223 8234246

[19] Cabrita LD, Dai WW, Bottomley SP (2004) Different conformational changes within the F-helix occur during serpin folding, polymerization, and proteinase lnhibition. Biochem 43: 9834–9839. 15274637 10.1021/bi0491346

[20] Baek J-H, Yang WS, Lee C, Yu M-H (2009) Functional Unfolding of α1-Antitrypsin Probed by Hydrogen-Deuterium Exchange Coupled with Mass Spectrometry. Molecular & Cellular Proteomics 8: 1072–1081. 19136720 10.1074/mcp.M800365-MCP200 PMC2689767

[21] James EL, Bottomley SP (1998) The mechanism of alpha(1)-antitrypsin polymerization probed by fluorescence spectroscopy. Arch Biochem and Biophys 356: 296–300. 9705220 10.1006/abbi.1998.0751

[22] Krishnan B, Gierasch LM (2011) Dynamic local unfolding in the serpin α-1 antitrypsin provides a mechanism for loop insertion and polymerization. Nat Struct Mol Biol 18: 222–226. 21258324 10.1038/nsmb.1976 PMC3074950

[23] Tew DJ, Bottomley SP (2001) Probing the equilibrium denaturation of the serpin alpha(1)-antitrypsin with single tryptophan mutants; Evidence for structure in the urea unfolded state. J Mol Biol 313: 1161–1169. 11700071 10.1006/jmbi.2001.5104

[24] Elliott PR, Abrahams J-P, Lomas DA (1998) Wild-type [alpha]1-antitrypsin is in the canonical inhibitory conformation. Journal of Molecular Biology 275: 419–425. 9466920 10.1006/jmbi.1997.1458

